# Sense of Smell in Individuals with Fibromyalgia: a Tractography Study

**DOI:** 10.1007/s00062-025-01515-6

**Published:** 2025-04-24

**Authors:** İlyas Uçar, Fatih Çiçek, Fatma Gül Ülkü Demir, Turgut Seber, Mehmet Hilmi Akdeniz, Ahmet Payas, Kerem Kökoğlu

**Affiliations:** 1 Department of Anatomy, Faculty of Medicine, Erciyes University, Kayseri, Turkey; 2 Department of Anatomy, Faculty of Medicine, Niğde Ömer Halis Demir University, Niğde, Turkey; 3 Department of Physical Medicine and Rehabilitation, Kayseri City Training and Research Hospital, University of Health Sciences, Kayseri, Turkey; 4 Department of Radiology, Kayseri City Training and Research Hospital, University of Health Sciences, Kayseri, Turkey; 5 Department of Otolaryngology, Faculty of Medicine, Erciyes University, Kayseri, Turkey; 6 Department of Anatomy, Faculty of Medicine, Amasya University, Amasya, Turkey

**Keywords:** Fibromyalgia, Sense of smell, MRI, Tractography

## Abstract

**Purpose:**

The etiopathogenesis of fibromyalgia (FM), which affects millions of people worldwide, is still debated. Recent research provides significant evidence that there are changes in the functions of the central and peripheral nervous systems and the sense of smell. This study analyzed the clinical assessment results of the sense of smell in individuals with FM and examined the olfactory-related structures in the nervous system.

**Methods:**

Thirty patients with FM and 31 age- and sex-matched asymptomatic controls participated in this cross-sectional study. Participants’ sense of smell was assessed with the Connecticut Chemosensory Clinical Research Center (CCCRC) including the Butanol threshold test (BET) and Smell Identification Tests. The total number of fibers, mean fiber length, the ratio of the number of fibers in this pathway to the number of fibers in the whole brain of the same individual, fractional anisotropy (FA), mean diffusion (MD), axial diffusion (AD) and radial diffusion (RD) values were calculated by tractography. Additionally, entorhinal cortex volume calculation was performed in MriStudio and MriCloud software using Diffusion tensor imaging (DTI) data in DICOM format.

**Results:**

The BET and CCCRC test scores were lower in the FM group (*p* < 0.05). Similarly, the mean FA values of the olfactory tract were lower on both the right and left sides in the FM group (*p* < 0.05). However, the entorhinal cortex volumes were similar, and there was no correlation between the right and left FA values of the olfactory tract and the BET scores or CCCRC scores in both groups (*p* > 0.05).

**Conclusion:**

Our study, which included participants’ self-assessments and data obtained from central nervous system (CNS) images, supports the idea that individuals with FM have a decreased olfactory function. Decreased FA values in individuals with FM may be an indicator of impaired myelin structure and axonal adaptation in individuals with FM.

## Key Points

- Our data shows that olfactory function is impaired in patients with fibromyalgia (FM).
- Tractography results demonstrate a reduction in FA (Fractional Anisotropy) values in FM patients compared to asymptomatic individuals.
- The decreased FA value may suggest disrupted myelin structure and axonal integrity in the olfactory pathways of the central nervous system in FM patients.

## Introduction

Fibromyalgia (FM) is a disorder characterised by chronic widespread pain with a low pain threshold. The main symptoms of FM are chronic widespread musculoskeletal pain or stiffness, typically accompanied by fatigue, sleep disturbances, anxiety, depression and memory problems. Although its etiology has not been fully elucidated, it is thought to be related to the central nervous system [1]. The prevalence of FM in the general population is 1.78% and mostly affects women aged 45–60 years [2].

The olfactory perception plays an important role in recognising the environment. Olfaction is a complex process CNS involving specific regions of the brain such as the limbic system. There is evidence of olfactory impairment in various neurological and psychiatric diseases such as Alzheimer’s disease, Down syndrome, Parkinson’s disease, schizophrenia and depression [3]. Reduced Olfactory Bulb volumes in patients with olfactory loss have been demonstrated by Magnetic Resonance Imaging (MRI) studies [4, 5].

Research shows that individuals with FM have impaired sense of smell. The sense of smell is closely related to the limbic system and the central nervous system, and therefore it is suggested that changes in FM may also affect the sense of smell [6, 7]. In the literature, there are studies reporting increased olfactory acuity in individuals with FM [6, 8]. However, a recent study reported a slight decrease in odour identification without a significant difference in odour threshold or discriminationi [9].

Studies reporting impaired olfactory function such as odour identification, odour threshold and discrimination among the symptoms of FM have been conducted only with olfactory tests. Since these tests consist of self-reported statements of patients, olfactory tests may be far from objective [6, 7, 9, 10]. Although there are many studies on the sense of smell in individuals with FM, studies examining the brain centres related to the sense of smell are limited [11].

MRI is a non-invasive imaging technology that produces detailed three-dimensional anatomical images using magnets and radio frequency waves [12]. Diffusion tensor imaging (DTI) technique, developed from the traditional MRI method, provides information about the microarchitecture of the tissue by measuring the diffusion rates and pathways of water molecules. Thus, it provides information about fibre length, fibre volume, fibre number and fibre conduction velocity of the pathways in the white matter of the human brain [13]. This method has become the preferred method in brain research to identify changes in fibres in the white matter [14].

Although there is evidence that there is a relationship between FM and sense of smell, there are conflicting statements [6, 9] and studies objectively evaluating olfactory sensitivity in individuals with FM are limited [11]. In this context, the aim of our study was to determine the microarchitecture of myelin and axon affecting fibre number, fibre thickness, fibre ratio and fibre conduction velocity of the olfactory tract carrying the sense of smell in FM and asymptomatic individuals by tractography method using brain DTI. In addition, to make recommendations in the light of reasonable and objective data by comparing with the data obtained from olfactory examination.

## Methods

### Study Design

Our study included 30 patients who were diagnosed with FM using the American College of Rheumatology (ACR) classification criteria between April 2024 and July 2024 by a specialist physician (FGUD) and 31 age- and sex-matched asymptomatic volunteers. Before starting the study, the purpose and scope of the study were explained to the participants in detail and informed consent forms were signed. The study was approved by the ethics committee (Date: 28.03.2024; Decision number: 2024/40) and conducted in accordance with the principles of the Declaration of Helsinki. The MRIs of the participants were evaluated pathologically by a radiologist (TS). Data of individuals with pathological findings were excluded from the study.

FM group (*n* = 30): Consists of female subjects aged 18–65 years with pain in at least 11 of 18 specific points on palpation for at least 3 months according to ACR diagnostic criteria.

Control group (*n* = 31): Asymptomatic female participants aged 18–65 years.

FM and control group exclusion criteria:

- Being previously diagnosed with FM and/or receiving treatment for FM.
- Having chronic systemic disease, inflammatory rheumatoid disease (rheumatoid arthritis, ankylosing spondylitis, systemic lupus erythematosus, etc.), history of autoimmune disorder with a history of malignancy or schizophrenia, pregnancy, breastfeeding and chronic smoking/alcohol/drug use.
- Having a chronic disease requiring the use of any neurological, psychiatric medication.
- Having any mental problem.
- History of sinonasal or anterior skull base surgery and nasal polyposis.
- History of otorhinolaryngology, including nasal endoscopy and/or imaging.
- Having Covid-19, having an active upper respiratory infection, being a smoker.

### Evaluation of the Sense of Smell

Both FM and asymptomatic participants were examined by otolaryngologist (KK and MHA). Participants who met the inclusion and exclusion criteria underwent the Connecticut Chemosensory Clinical Research Center (CCCRC). The two-stage odor test was performed by investigators who did not know which group the participants were in. The first stage of the odor test consisted of the Butanol threshold test (BET) and the second stage consisted of the Smell Identification Test.

*BET:* 7 50 cc glass bottles were filled with 4, 1, 0.4, 0.1%, 0.1, 0.05, 0.01 and 0.005% concentrations of N‑Butanol and distilled water. Participants were presented with the solutions to smell from the most dilute to the most concentrated solution and the bottle they smelled was determined as the threshold. The participant had to select the same bottle 2 times to be determined as the threshold odor bottle. The participant was blindfolded during the test. The test was applied to the nasal passages separately. According to the test result, participants received scores between 0–7. Both passage scores were averaged.

*Smell Identification Test:* Vicks, mothballs, chocolate, cinnamon, coffee, peanut butter, baby powder and soap were in 8 50 cc glass bottles with no visible inside. The average room temperature was between 20–25 °C. Before the test, the participants examined a visual and written list of 16 odor sources and were asked to identify the odor of the bottle from the list of odors. The list included peanuts, black pepper, baby powder, cigarettes, soap, rubber, coffee, mothballs, cinnamon, wood shavings, chocolate, garlic, ketchup, fish, onion, Vicks. Vicks odor was identified by all participants. It was used as a determinant odor for olfactory function ability and was not included in the scoring. One point was awarded for each correctly identified odor. The test was applied to the nasal passages separately and the average of the two nasal passages was taken.

*CCCRC Score:* While calculating the CCCRC score, the mean of the participants’ BET and Smell Identification Test scores were taken. According to the test score, participants were classified as normosmia (6–7), mild hyposmia (5–5.75), moderate hyposmia (4–4.75), severe hyposmia (2–3.75) and anosmia (0–1.75) between 0–7 points.

### Acquisition of MR Images

A 3 T (Tesla) Siemens Magnetom Skyra, Netherlands brand MRI device was used to acquire the volunteers. T1 magnetization-prepared rapid acquisition with gradient echo (MPRAGE) sequence was used to evaluate anatomical structures and fluid attenuated inversion recovery (FLAIR) sequence was used to evaluate brain anomalies. In addition, DTI was performed to visualize possible differences in brain regions.

### MRI Protocol

- T1 weighted MPRAGE sequence: Sagittal, Repetition time (TR) = 2300 ms, Echo Time (TE) = 3.4 ms, FOV = 250 mm, Matrix:256 × 256, Slice Thickness = 1 mm.
- DTI: Axial, TR = 4900 ms, TE = 95 ms, Number-of-Slice = 36, FOV = 230 mm, Matrix: 128 × 128, Slice Thicknes = 3.5 mm, Averages = 3, b = 0.1000 s/mm2, 20 diffusion directions.
- FLAIR sequence: Axial, TR = 9000, TE = 87, TI = 2500, FA = 150, FOV = 240 mm, Slice Thickness = 5 mm.

### Image Processing

#### Tractography Analysis of the Olfactory Tract

Tractography of the targeted pathways from the brain DTIs of the participants was performed in the DSI Studio program downloaded from http://dsi-studio.labsolver.org/. Before starting the tractography process, in the “Fiber tracking” tab; “Threshold” was set to 0.20, “Angular Threshold” was set to 70 degrees, “Smoothing” was set to 0.50, the shortest tract was set to 10 mm, the longest tract was set to 1000 mm, and “terminateif” was set to 100,000 fibers. In each new image, these parameters were adjusted again before starting the tractography procedure. In our study, olfactory tract was examined bilaterally. The total number of fibers, mean fiber length (in millimeters), the ratio of the number of fibers in this pathway to the number of fibers in the whole brain of the same individual (fiber ratio = number of fibers in the pathway*100/number of fibers in the whole brain), fractional anisotropy (FA), mean diffusion (MD), axial diffusion (AD) and radial diffusion (RD) values were calculated.

#### Volume Analysis of the Entorhinal Cortex

In the study, entorhinal cortex volume calculation was performed in MriStudio and MriCloud software using DTI data in DICOM format. These programs can be accessed by logging in (registration) with username and password via the website “https://MriStudio.org”. After the procedures, a parcellation map was opened on the images to be parcellated and images of 168 regions of the brain were obtained. From these regions, the volume of the entorhinal cortex was obtained in cm^3^ and recorded.

### Statistical Analysis

The normal distribution of the measurements obtained from the study groups was evaluated by Shapiro-Wilks test. Independent‑t test was used to compare the two groups according to the distribution of the measured parameters. Pearson Correlation test was used to examine the relationship between numerical variables. Statistically *p* < 0.05 was considered significant. Statistical analysis of the data obtained from the images was performed in SPSS 25.0 (SPSS, Inc., Chicago, IL, USA).

## Results

The comparison of scores obtained from olfactory tests, tractography analysis results of tr. olfactorius and volume measurement results of entorhinal cortex between FM and asymptomatic control group individuals are summarized in Table 1. Accordingly, age and Body mass index (BMI) values were similar in both groups (*p* > 0.05). BET and CCCRC test scores, which we used to test the sense of smell, were lower in the FM group (*p* < 0.05). Similarly, the mean FA values of tr. olfactorius were lower in the FM group on both right and left sides (*p* < 0.05) (Table 1).

**Table 1:** Comparison of measured parameters between FM and control group

Parameters	Control (*N* = 31)		FM (*N* = 30)		*p*
	Mean ± S.D	Min-Max	Mean ± S.D	Min-Max	
Age (years)	43.68 ± 10.13	24.00–68.00	46.80 ± 8.01	24.00–63.00	0.188
BMI	26.43 ± 4.07	18.33–35.38	28.23 ± 3.99	17.41–35.15	0.088
BET	6.18 ± 1.61	0.00–7.00	4.85 ± 1.77	2.00–7.00	*0.003*
Smell identification test	5.52 ± 1.55	0.00–7.00	4.68 ± 1.78	0.00–7.00	0.056
CCCRC	5.85 ± 1.52	0.00–7.00	4.78 ± 1.63	1.00–7.00	*0.010*
Olfactory Tract number (R)	5792.87 ± 1519.65	3656.00–8329.00	5560.87 ± 1946.02	2452.00–9339.00	0.605
Olfactory Tract FA (R)	0.37 ± 0.03	0.28–0.42	0.36 ± 0.03	0.30–0.42	*0.020*
Olfactory Tract number (L)	3683.77 ± 1826.68	1283.00–8329.00	3917.63 ± 2306.24	1326.00–10,253.00	0.662
Olfactory Tract FA (L)	0.35 ± 0.03	0.28–0.40	0.33 ± 0.03	0.27–0.39	*0.005*
Entorhinal Cortex Volume (cm^3^)	3.95 ± 0.39	3.35–5.02	3.95 ± 0.45	3.18–4.94	0.989
Entorhinal Cortex Volume (R) (cm^3^)	2.00 ± 0.20	1.59–2.48	2.03 ± 0.27	1.51–2.50	0.609
Entorhinal Cortex Volume (L) (cm^3^)	1.95 ± 0.24	1.57–2.65	1.92 ± 0.23	1.54–2.57	0.596

*FM* Fibromyalgia, *BMI* Body mass index, *BET* Butanol threshold test, *CCCRC* Connecticut Chemosensory Clinical Research Center, *FA* Fractional anisotropy

The results of the correlation analysis between olfactory tract and BET and CCCRC smell tests are summarized in Table 2. Accordingly, no correlation was found between the right and left FA values of tr. olfactorius and BET score and CCCRC score in FM and control groups (*p* > 0.05). However, there was a very high positive linear relationship between BET score and CCCRC score in both groups (*p* < 0.001; r = 0.904; r = 0.963). In addition, there was a moderate positive linear relationship between FA values of right and left tr. olfactorius in both groups (*p* < 0.05; r = 0.491; r = 0.647).

**Table 2:** Correlation values between the FA value of tr olfactorius and BET and CCCRC odor tests between FM and control group

Group	Parameters		CCCRC	Olfactory Tract FA (R)	Olfactory Tract FA (L)
FM	BET	r	0.904^*^	0.066	−0.072
		*p*	*0*.*000*	0.729	0.705
		*N*	30	30	30
	CCCRC	r	1	0.062	−0.066
		*p*	–	0.746	0.730
		*N*	–	30	30
	Olfactory Tract FA (R)	r	–	1	0.491^*^
		*p*	–	–	*0.006*
		*N*	–	–	30
Control	BET	r	0.963^*^	−0.146	0.003
		*p*	*0.000*	0.435	0.987
		*N*	31	31	31
	CCCRC	r	1	−0.179	−0.076
		*p*	–	0.337	0.685
		*N*	–	31	31
	Olfactory Tract FA (R)	r	–	1	0.647^*^
		*p*	–	–	*0.000*
		*N*	–	–	31

*FM* Fibromyalgia, *BMI* Body mass index, *BET* Butanol threshold test, *CCCRC* Connecticut Chemosensory Clinical Research Center, *FA* Fractional anisotropy, *r* Correlation coefficient between two variables, *p* Significant value, *N* Sample size

The mean scores obtained from the odor tests performed on FM and asymptomatic control group are shown in Table 1. The mean BET test score was 4.85 ± 1.77 in the FM group and 6.18 ± 1.61 in the control group (*p* < 0.05). Smell Identification Test score was 4.68 ± 1.78 in the FM group and 5.52 ± 1.55 in the control group (*p* > 0.05). CCCRC score was 4.78 ± 1.63 and 5.85 ± 1.52 in FM and control groups, respectively (*p* < 0.05).

## Dıscussion

In the present study, Olfactory Tract fibers and Entorhinal Cortex volumes of individuals with FM were determined and compared with olfactory examination results. Our data showed that individuals with FM had impaired sense of smell and decreased Olfactory Tract FA values. Compared to asymptomatic individuals, the Olfactory Tract fiber count and Entorhinal Cortex volume values were similar in individuals with FM and there was no difference in odor discrimination.

In studies comparing individuals with FM with healthy individuals, differences in brain activation, changes in neuronal transmitters and anatomical differences in brain structure have been reported [15, 16]. Considering the various comorbidities of FM with psychiatric conditions such as depression, anxiety and stress-related disorders, it has been reported that olfactory disorders can also be expected [10]. Therefore, in our study, we performed olfactory examination of individuals with FM and determined the Olfactory Tract fibers and the volume of the Entorhinal Cortex in the cortex where the sense of smell is processed by tractography method.

There are many variables that affect olfactory function. There are studies reporting that women perform better than men, olfactory acuity decreases in the elderly, smoking alters olfactory function, and odor perception decreases after Covid-19 infection [17–19]. In addition, FM syndrome is a disorder mostly seen in women between the ages of 45–60 [2]. In order to minimize the effect of these variables on the results, our study included age- and BMI-matched, non-smokers, women without a history of Covid-19 infection, and women diagnosed with FM for the first time.

The sense of smell begins with the conversion of chemical substances detected by olfactory receptors in the nasal cavity into nerve signals. These signals are transmitted to the brain via olfactory nerves and processed in the limbic system. The sense of smell plays an important role in many cognitive and emotional processes such as memory, emotional responses and food preferences [20]. Therefore, smell tests can be helpful in confirming the disease occurring in the person, determining its degree, revealing those who are not really sick and in terms of the prognosis of the disease [21]. There are generally two subgroups of odor tests: The first are psychophysical tests and the second are electrophysiological tests. While psychophysical tests are used for clinical evaluation of smell loss, electrophysiological tests are primarily used for research purposes [22]. However, psychophysical tests were mostly used in studies investigating the relationship between FM and sense of smell due to their ease of application [6–9]. In our study, we evaluated the participants’ sense of smell with the CCCRC, which consists of two stages, the BET and the Smell Identification Test. Our data showed that individuals with FM had impaired sense of smell perception in the Smell Identification Test (*p* = 0.056) with no difference in BET (*p* = 0.003) and CCCRC (*p* = 0.010) scores compared to asymptomatic individuals. In the literature, there are studies reporting increased olfactory acuity in individuals with FM [6, 8], no significant difference in odor threshold or discrimination, but a slight decrease in odor identification [7, 9]. The reason for these contradictory statements may be due to the psychophysical tests used in the clinical evaluation of odor loss, which include subjective data. Therefore, it may be important to perform electrophysiologic tests or simultaneous imaging of olfactory structures in the brain in order to make an absolute assessment of the relationship between FM and odor.

Thanks to the DTI technique developed from the MRI method, it is possible to obtain information about fiber length, fiber volume, fiber number and fiber conduction velocity of the pathways in the white matter of the human brain [13]. This method, which has been preferred in brain research in recent years, has an increasing popularity [14, 23, 24]. In the present study, both right olfactory tract FA (*p* = 0.020) and left olfactory tract FA (*p* = 0.005) were lower in individuals with FM compared to asymptomatic individuals. DTI evaluates the direction of diffusion and the degree of anisotropy. It provides information about microstructural changes in the brain by measuring the diffusive motion of water molecules. In DTI analysis, several coefficients are used to calculate the diffusion tensor of interest [25]. The most commonly used are FA, MD, AD and RD. The most common of these indicators is FA. FA is a measure of the overall anisotropy of the tissue and has values between 0 and 1. It is often interpreted as an indicator of white matter integrity. A high FA value indicates high white matter integrity, while a low FA value indicates impaired white matter integrity [26]. These data support the idea that individuals with FM may have impaired olfactory perception and anatomical differences in brain structure.

In an imaging study conducted by Sayılır et al., olfactory bulb volumes of patients diagnosed with FM were calculated and it was reported that the FM group had a lower olfactory bulb volume [11]. In addition to tractography, we also calculated the volumes of the entorhinal cortex, the cortex region where the sense of smell is processed. Our data revealed that the entorhinal cortex volumes were similar to the control group (*p* = 0.989).

In our study, no correlation was found between the participants’ right and left FA values of the Olfactory Tract and the psychophysical tests used for the clinical evaluation of odor loss. There may be two reasons for this. First, since our study group consisted of individuals who had just been diagnosed with FM, the physical examination findings of the patient may not yet be reflected in the CNS. Secondly, patients may have exaggerated their odor sensitivity. Researchers should keep in mind that not every clinical finding may be reflected in the image and not every finding in the image may be reflected in the clinic.

No correlation was found between BET score and CCCRC score (*p* > 0.05). However, a very high positive linear relationship was found between BET score and CCCRC score in both groups (*p* < 0.001; r = 0.904; r = 0.963).

### Limitations

Our study has several limitations. One of the limitations of the current study is that the educational or socioeconomic differences of the participants should be taken into account considering their potential to influence the results. These factors have been reported to be associated with FM [27]. Secondly, we included only female subjects in our study, considering that FM affects mostly women. Therefore, transferring clinical implications to male patients may be misleading. Finally, our data were obtained from a small sample. Therefore, the results should be taken with caution.

## Conclusion

The relationship between FM and the sense of smell is an important area of research to understand the pathophysiology of this syndrome and to improve the quality of life of individuals with FM. This study, which includes participants’ self-assessments and data from CNS images, supports the idea that individuals with FM have a reduced olfactory function. Decreased FA in individuals with FM may be indicative of impaired myelin structure and axonal cohesion in individuals with FM. Future longitudinal studies with larger sample sizes are needed to examine the effects of FM on the sense of smell over time. In addition, studies evaluating the effects of FM treatments on the sense of smell should be conducted.

## References

[1] Kocyigit BF, Akyol A. Coexistence of fibromyalgia syndrome and inflammatory rheumatic diseases, and autonomic cardiovascular system involvement in fibromyalgia syndrome. Clin Rheumatol. 2023;42(3):645–52. 10.1007/s10067-022-06385-8. 36151442 10.1007/s10067-022-06385-8

[2] Blanco S, Sanromán L, Pérez-Calvo S, Velasco L, Peñacoba C. Olfactory and cognitive functioning in patients with fibromyalgia. Psychol Health Med. 2019;24(5):530–41. 10.1080/13548506.2018.1549741. 30453770 10.1080/13548506.2018.1549741

[3] Perricone C, Shoenfeld N, Agmon-Levin N, de Carolis C, Perricone R, Shoenfeld Y. Smell and autoimmunity: a comprehensive review. Clinic Rev Allerg Immunol. 2013;45:87–96. 10.1007/s12016-012-8343-x. 10.1007/s12016-012-8343-x 23233263

[4] Rombaux P, Duprez T, Hummel T. Olfactory bulb volume in the clinical assessment of olfactory dysfunction. Rhinology. 2009;47(1):3. 10.1038/s41598-023-36783-0. 19382487

[5] Mueller A, Abolmaali ND, Hakimi AR, Gloeckler T, Herting B, Reichmann H, Hummel T. Olfactory bulb volumes in patients with idiopathic parkinson’s disease a pilot study. J Neural Transm. 2005;112:1363–70. 10.1007/s00702-005-0280-x. 15711853 10.1007/s00702-005-0280-x

[6] Dorris ER, Maccarthy J, Simpson K, McCarthy GM. Sensory perception quotient reveals visual, scent and touch sensory hypersensitivity in people with fibromyalgia syndrome. Front Pain Res. 2022;3:926331. 10.3389/fpain.2022.926331. 10.3389/fpain.2022.926331 PMC9294149 35866137

[7] Amital H, Agmon-Levin N, Shoenfeld N, Arnson Y, Amital D, Langevitz P, Shoenfeld Y. Olfactory impairment in patients with the fibromyalgia syndrome and systemic sclerosis. Immunol Res. 2014;60:201–7. 10.1007/s12026-014-8573-5. 25424576 10.1007/s12026-014-8573-5

[8] Demarquay G, Ryvlin P, Royet JP. Olfaction and neurological diseases: a review of the literature. Revue Neurol. 2007;163(2):155–67. 10.1016/s0035-3787(07)90387-2. 10.1016/s0035-3787(07)90387-2 17351535

[9] Wilbarger JL, Cook DB. Multisensory hypersensitivity in women with fibromyalgia: implications for well being and intervention. Arch Phys Med Rehabil. 2011;92(4):653–6. 10.1016/j.apmr.2010.10.029. 21440712 10.1016/j.apmr.2010.10.029 PMC3272297

[10] Lötsch J, Kraetsch HG, Wendler J, Hummel T. Self-ratings of higher olfactory acuity contrast with reduced olfactory test results of fibromyalgia patients. Int J Psychophysiol. 2012;86(2):182–6. 10.1016/j.ijpsycho.2012.09.003. 22985737 10.1016/j.ijpsycho.2012.09.003

[11] Sayılır S, Çullu N. Decreased olfactory bulb volumes in patients with fibromyalgia syndrome. Baillieres Clin Rheumatol. 2017;36:2821–4. 10.1007/s10067-017-3772-9. 10.1007/s10067-017-3772-9 28744789

[12] Mathew AJ, Bird P, Gupta A, George R, Danda D. Magnetic resonance imaging (MRI) of feet demonstrates subclinical inflammatory joint disease in cutaneous psoriasis patients without clinical arthritis. Clin Rheumatol. 2018;37:383–8. 10.1007/s10067-017-3895-z. 29204762 10.1007/s10067-017-3895-z

[13] Jang SH, Kwon YH, Lee MY, Kim JR, Seo JP. Aging of the cingulum in the human brain: preliminary study of a diffusion tensor imaging study. Neurosci Lett. 2016;610:213–7. 10.1016/j.neulet.2015.11.018. 26598020 10.1016/j.neulet.2015.11.018

[14] Cheng H, Wang Y, Sheng J, Kronenberger WG, Mathews VP, Hummer TA, Saykin AJ. Characteristics and variability of structural networks derived from diffusion tensor imaging. Neuroimage. 2012;61(4):1153–64. 10.1016/j.neuroimage.2012.03.036. 22450298 10.1016/j.neuroimage.2012.03.036 PMC3500617

[15] Burgmer M, Pfleiderer B, Maihöfner C, Gaubitz M, Wessolleck E, Heuft G, Pogatzki-Zahn E. Cerebral mechanisms of experimental hyperalgesia in fibromyalgia. Eur J Pain. 2012;16(5):636–47. 10.1002/j.1532-2149.2011.00058.x. 22337349 10.1002/j.1532-2149.2011.00058.x

[16] Schweinhardt P, Sauro KM, Bushnell MC. Fibromyalgia: a disorder of the brain? Neuroscientist. 2008;14(5):415–21. 10.1177/1073858407312521. 18270311 10.1177/1073858407312521

[17] Frye RE, Schwartz BS, Doty RL. Dose-related effects of cigarette smoking on olfactory function. JAMA. 1990;263(9):1233–6. 10.1001/jama.1990.03440090067028. 2304239

[18] Hummel T, Lötsch J. Prognostic factors of olfactory dysfunction. Arch Otolaryngol Neck Surg. 2010;136(4):347–51. 10.1001/archotol.125.10.1071. 10.1001/archoto.2010.27 20403850

[19] Di Stadio A, Brenner MJ, De Luca P, Albanese M, D’Ascanio L, Ralli M, Bernitsas E. Olfactory dysfunction, headache, and mental clouding in adults with long-COVID-19: what is the link between cognition and olfaction? A cross-sectional study. Brain Sci. 2022;12(2):154. 10.3390/brainsci12020154. 35203918 10.3390/brainsci12020154 PMC8870047

[20] Hadley K, Orlandi RR, Fong KJ. Basic anatomy and physiology of olfaction and taste. Otolaryngol Clin North Am. 2004;37(6):1115–26. 10.1016/j.otc.2004.06.009. 15563905 10.1016/j.otc.2004.06.009

[21] Wrobel BB, Leopold DA. Clinical assessment of patients with smell and taste disorders. Otolaryngol Clin North Am. 2004;37(6):1127–42. 10.1016/j.otc.2004.06.010. 15563906 10.1016/j.otc.2004.06.010 PMC7118991

[22] Cao Z, Yang A, D’Aloisio AA, Suarez L, Deming-Halverson S, Li C, Chen H. Assessment of self-reported sense of smell, objective testing, and associated factors in middle-aged and older women. Jama Otolaryngol Neck Surg. 2022;148(5):408–17. 10.1001/jamaoto.2022.0069. 10.1001/jamaoto.2022.0069 PMC8914911 35266981

[23] Payas A, Batin S, Kurtoğlu E, Arik M, Seber T, Uçar İ, Unur E. Is the integration problem in the sensoriomotor system the cause of adolescent idiopathic scoliosis? J Pediatr Orthop. 2023;43(2):111–9. 10.1097/BPO.0000000000002300. 36418290 10.1097/BPO.0000000000002300

[24] Kurtoğlu E, Payas A, Düz S, Arık M, Uçar I, Tokmak TT, Unur E. Analysis of changes in brain morphological structure of taekwondo athletes by diffusion tensor imaging. J Chem Neuroanat. 2023;129:102250. 10.1016/j.jchemneu.2023.102250. 36791923 10.1016/j.jchemneu.2023.102250

[25] Tournier JD, Mori S, Leemans A. Diffusion tensor imaging and beyond. Magn Reson Med. 2011;65(6):1532. 10.1002/mrm.22924. 21469191 10.1002/mrm.22924 PMC3366862

[26] Hagmann P, Jonasson L, Maeder P, Thiran JP, Wedeen VJ, Meuli R. Understanding diffusion MR imaging techniques: from scalar diffusion-weighted imaging to diffusion tensor imaging and beyond. Radiographics. 2006;26(1):205–23. 10.1148/rg.26si065510. 10.1148/rg.26si065510 17050517

[27] Bergman S. Psychosocial aspects of chronic widespread pain and fibromyalgia. Disabil Rehabil. 2005;27(12):675–83. 10.1080/09638280400009030. 16012060 10.1080/09638280400009030

